# GP2a I118 and GP4 D43 play critical roles in the attachment of PRRSV to the CD163 receptor: implications for anti-PRRSV infection targets

**DOI:** 10.1128/jvi.00963-25

**Published:** 2025-08-18

**Authors:** Guoqing Liu, Xinyi Huang, Yongbo Yang, Meng Chen, Xiaoxiao Tian, Hao Song, Haojie Wang, Shujie Wang, Haiwei Wang, Xuehui Cai, Tongqing An

**Affiliations:** 1 State Key Laboratory for Animal Disease Control and Prevention, Harbin Veterinary Research Institute, Chinese Academy of Agricultural Sciences 111613 , Harbin, China; 2 Heilongjiang Provincial Key Laboratory of Veterinary Immunology, Harbin, China; 3 Heilongjiang Veterinary Biopharmaceutical Engineering Technology Research Center, Harbin, China; University of Kentucky College of Medicine, Lexington, Kentucky, USA

**Keywords:** PRRSV, cellular tropism, CD163 receptor, blocking peptide

## Abstract

Porcine reproductive and respiratory syndrome virus (PRRSV) poses a significant threat to the global swine industry. Numerous modified live vaccines (MLVs) against PRRSV have been developed through the serial passage of wild-type parental strains in Marc-145 cells. However, the infectivity of these MLVs toward their primary target cell *in vivo*, porcine alveolar macrophage (PAM), is markedly reduced. The underlying mechanism for this decreased tropism remains unclear. In this study, we determined that the 118th residue in GP2a and the 43rd residue in GP4 play critical roles in determining viral tropism for PAM cells. Individual or combined mutations of GP2a-V118I and GP4-N43D significantly enhanced viral attachment to PAM cells. Furthermore, synthesized small peptides containing GP2a-I118 and GP4-D43 effectively blocked viral attachment to PAM cells in a dose-dependent manner, exhibiting broad-spectrum blocking effects against various PRRSV strains, including newly emerged NADC30- and NADC34-like PRRSVs. Coimmunoprecipitation (co-IP) results demonstrated that mutations or deletions at GP2a-V118 and GP4-N43 could significantly affect the binding affinity of GP2a and GP4 for the CD163 receptor, particularly the SRCR5 domain of CD163. Overall, for the first time, we identified two key residues associated with the reduced infectivity of PRRSV MLVs, which play essential roles in the attachment of PRRSV to the CD163 receptor. These findings provide novel insights into PRRSV tropism for PAM cells and reveal new targets for the development of peptide-based drugs or neutralizing antibodies against PRRSV infection.

**IMPORTANCE:**

Currently, most modified live vaccines (MLVs) against animal diseases are derived from serial passages of parental virulent viruses in heterologous animal cells. This process enhances viral adaptation to heterologous cells while significantly reducing viral infectivity to host animal cells, thereby attenuating virulence in hosts. However, the mechanisms underlying the changes in tropism of many MLVs remain largely unknown. In this study, we identified and confirmed two key residues associated with changes in tropism. Importantly, we demonstrated that small peptides can block viral binding to receptors. These findings not only provide potential targets for the development of antiviral drugs or neutralizing antibodies but also offer valuable references for studying tropism changes in other viruses.

## INTRODUCTION

Porcine reproductive and respiratory syndrome (PRRS) is caused by the PRRS virus (PRRSV), which primarily induces reproductive disorders in sows and respiratory diseases in piglets and growing pigs, resulting in an estimated annual loss of $2.7 billion to the global swine industry (1). PRRSV is a single-stranded positive-sense RNA virus belonging to the genus *Betaarterivirus*, family *Arteriviridae*, and order *Nidovirales*. It comprises two species: *Betaarterivirus suid* 1 (PRRSV-1, formerly known as European-type PRRSV) and *Betaarterivirus suid* 2 (PRRSV-2, formerly known as North American-type PRRSV) (2). Its genome is approximately 15 kb in length and contains at least 10 open reading frames (ORFs), encoding at least 14 nonstructural proteins (NSPs) and eight structural proteins. Among these proteins, the nonglycosylated structural protein E is encoded by ORF2b, whereas the minor glycoproteins GP2a and GP4 are encoded by ORF2a and ORF4, respectively (3).

PRRSV exhibits restricted host and cellular tropism, with porcine alveolar macrophage (PAM) being the primary target cells *in vivo. In vitro*, it can infect African green monkey kidney cell lines (MA-104) and their derivative cell line (Marc-145) (4). PRRSV infection involves multiple cellular receptors, including heparan sulfate (5), sialoadhesin (CD169) (6), CD163 (7), CD151 (8), DC-SIGN (CD209) (9), vimentin (10), and MYH9 (11). Among these, the scavenger receptor CD163 is indispensable for viral entry (12). Transfection of CD163 into nonsusceptible cell lines confers PRRSV susceptibility, and stable CD163-expressing cells permit viral replication and progeny production (7, 13). PRRSV enters cells via clathrin-mediated endocytosis and macropinocytosis, with CD163 serving as a critical mediator in both pathways (14, 15). The fifth scavenger receptor cysteine-rich (SRCR5) domain of CD163 has been identified as the primary binding site for PRRSV (16). CD163-specific antibodies recognizing SRCR5 can reduce PRRSV infection (16). Gene-edited pigs lacking the CD163 SRCR5 domain are resistant to PRRSV infection (17). Blocking the interaction between PRRSV and CD163-SRCR5 via small-molecule compounds significantly inhibits PRRSV infection of PAM cells (18). The tropism of PRRSV is reported to be associated with the minor structural proteins E, GP2a, GP3, and GP4 (19, 20). CD163 facilitates PRRSV attachment and internalization by interacting with viral GP2a and GP4 (7, 21). Furthermore, substitutions at amino acid residues 91, 97, and 98 in GP2a significantly increase PRRSV replication in Marc-145 cells (22, 23). PRRSV infects PAM or Marc-145 cells via distinct receptors. Notably, serial passage in Marc-145 cells reduces the pathogenicity of PRRSV in pigs. Consequently, more than 10 commercial PRRSV modified live vaccines (MLVs) have been derived from serial passages in Marc-145 cells and have been widely used globally for over 20 years (4). However, PRRSV MLVs cannot effectively replicate in PAM cells (24). For example, after 95 passages in Marc-145 cells, PRRSV HCV1 lost its ability to infect PAM cells (25). The underlying mechanism for the reduced infectivity of MLV strains toward PAM cells, the target cells *in vivo*, remains unclear.

In this study, two amino acid mutations (GP2a I118V and GP4 D43N) in highly pathogenic PRRSV (HP-PRRSV) MLVs were identified as responsible for the loss of infectivity to PAM cells. Furthermore, infection of wild-type PRRSV in PAM cells could be blocked by small-molecule peptides containing these two amino acids. These findings suggest that these two amino acids play crucial roles in PRRSV tropism, providing novel targets for neutralizing antibodies or antiviral drugs.

## RESULTS

### HP-PRRSV MLV strains lose infectivity to PAM cells

**Fig 1:**
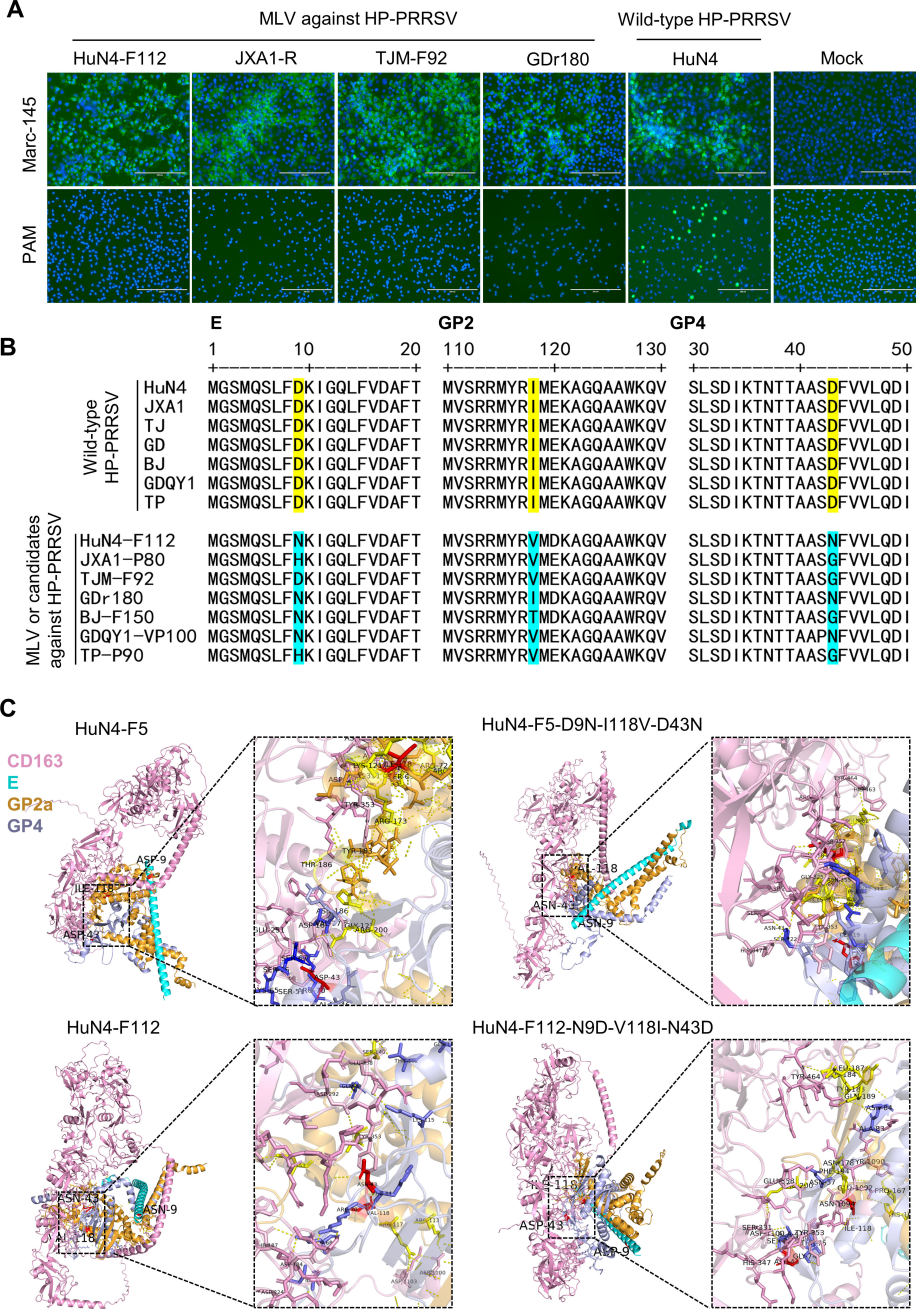
Infectivity of HP-PRRSV MLVs in PAM cells and sequence comparison between MLVs and their parental strains. (**A**) IFA detection of PAMs and Marc-145 cells infected with four HP-PRRSV MLVs or wild-type PRRSV HuN4 at an MOI of 0.01. A monoclonal antibody (1B4) targeting the N protein was used to label the PRRSV. Scale bars: 200 µm. (**B**) Sequence alignment of HP-PRRSV MLVs and their parental strains. Three amino acid residues in E, GP2a, and GP4 with relatively high mutation frequencies are highlighted. (**C**) A close-up view of the interfaces of GP2a, GP4, and CD163 is shown. The amino acid residues within GP2a and GP4 that interact with CD163 via hydrogen bonds were labeled.

Four commercial HP-PRRSV MLV strains (HuN4-F112, JXA1-R, GDr180, and TJM-F92) were used to infect Marc-145 or PAM cells at a multiplicity of infection (MOI) of 0.01. Immunofluorescence assay (IFA) results revealed that serial passaging of Marc-145 cells enabled these vaccine strains to acquire efficient replication capacity but resulted in either complete loss or a significant reduction in their ability to infect PAM cells (Fig. 1A). Comparative genomic analysis of seven commercial PRRSV MLV or MLV candidate strains (HuN4-F112, JXA1-R, GDr180, TJM-F92, BJ-F150, GDQY1-VP100, and TP-P90) and their corresponding wild-type parental strains revealed high-frequency mutation sites in the E, GP2a, and GP4 proteins. Notably, typical mutations, such as D9N/H in E, I118V in GP2a, and D43N/G in GP4, were detected in the vaccine strains (Fig. 1B), suggesting their potential involvement in altered cellular tropism. On the basis of AlphaFold 3 predictions, the complex structure of GP2a, GP3, GP4, and the CD163 receptor was generated. HuN4-F5 and HuN4-F112 were compared with their mutant strains. Hydrogen bonds and salt bridges were observed in the interaction between the CD163 receptor and the GP2a/GP4 proteins. Notably, compared with HuN4, the I118V mutation in GP2a and the D43N mutation in GP4 of HuN4-F5-D9N-I118V-D43N decreased hydrogen bonding with the CD163 receptor. The hydrogen bonding between GP2a/GP4 and CD163 was also greater in the HuN4-F112-N9D-V118I-N43D mutant than in the original HuN4-F112 strain (Fig. 1C). Moreover, in the HuN4-F112 strain, the 118th amino acid of GP2a and the 43rd amino acid residue of GP4 (red) are exposed on the surface of the proteins.

### Rescue of site-directed mutants of HuN4-F5 or HuN4-F112

**Fig 2:**
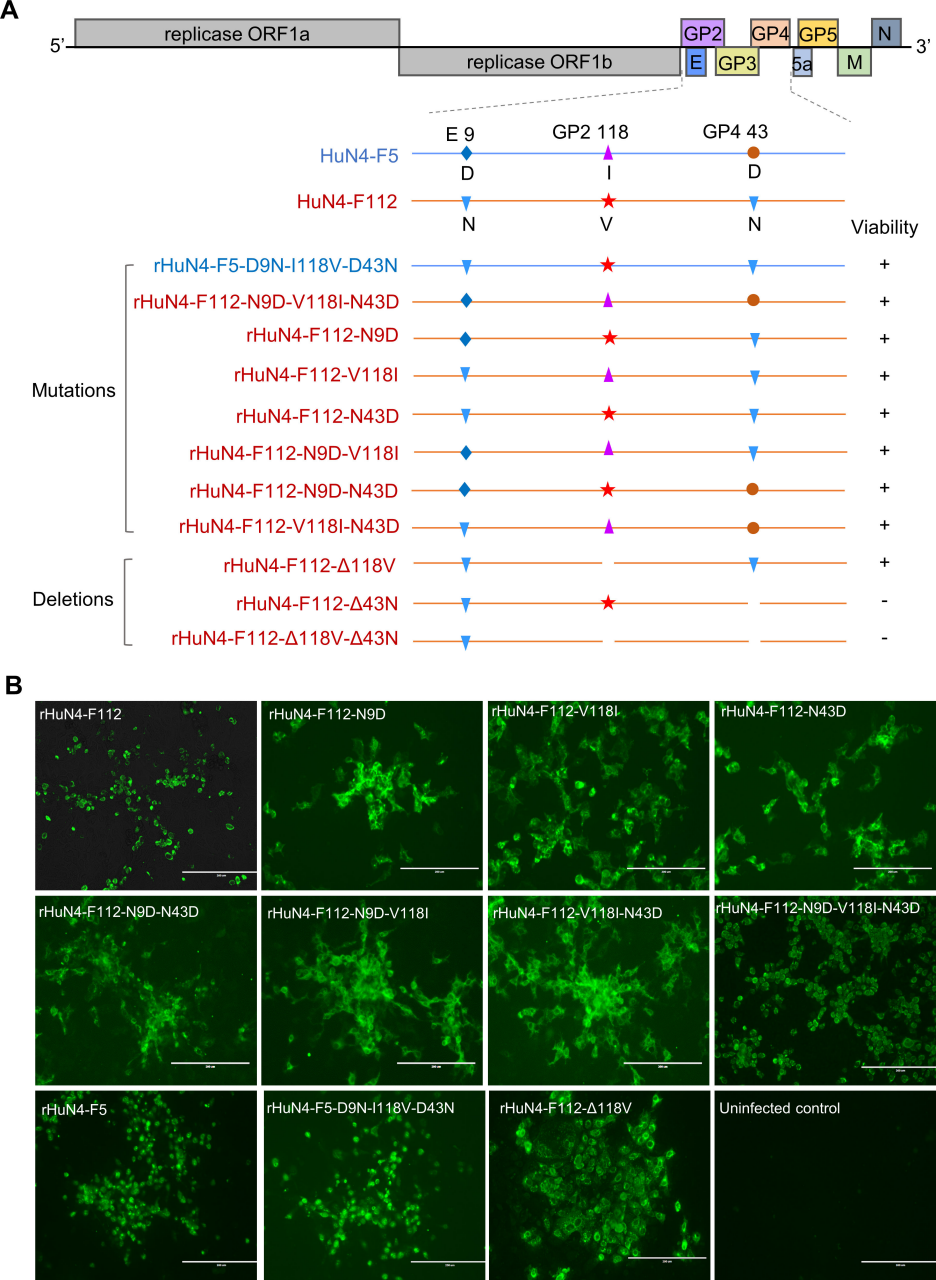
Construction and rescue of PRRSV mutants. (**A**) Schematic illustration of the construction of mutant and deletion viruses based on HuN4-F5 and HuN4-F112 infectious clones. (**B**) IFA identification of the rescued viruses. Infectious clone plasmids of the mutant viruses were transfected into Marc-145 cells. At 6 days post-transfection, IFA was performed with the PRRSV monoclonal antibody to identify the rescued viruses.

To determine whether these three amino acid mutations affect the viral tropism of PAM cells, a series of single or multiple site-directed mutants at positions 9, 118, and 43 in E, GP2a, and GP4 were constructed on the basis of the HuN4-F5 and HuN4-F112 infectious clones, respectively. Using the HuN4-F5 infectious clone as the backbone, a three-point mutant virus, rHuN4-F5-D9N-I118V-D43N, was constructed. Using the HuN4-F112 infectious clone as the backbone, three-point mutant virus rHuN4-F112-N9D-V118I-N43D, two-point mutant viruses rHuN4-F112-N9D-V118I, rHuN4-F112-N9D-N43D and rHuN4-F112-V118I-N43D, and single-point mutant viruses rHuN4-F112-N9D, rHuN4-F112-V118I, and rHuN4-F112-N43D were constructed. Additionally, on the basis of the HuN4-F112 infectious clone, deletion variants of GP2a residue 118 and GP4 residue 43, either individually or in combination, were generated (Fig. 2A).

The IFA results demonstrated that introducing D9N, I118V, and D43N mutations into HuN4-F5 yielded mutant viruses; similarly, introducing N9D, V118I, and N43D mutations into HuN4-F112, either individually or in combination, produced infectious viruses (Fig. 2B). Moreover, the GP2a-118V-deleted rHuN4-F112 remained viable, indicating that residue V118 is dispensable for viral particle infectivity. However, mutants with GP4 N43 deletion could not be rescued, confirming that GP4 N43 is important for producing infectious progeny viruses.

### HuN4-F112 MLV-based GP2a or GP4 revertants restore proliferation in PAM cells

**Fig 3:**
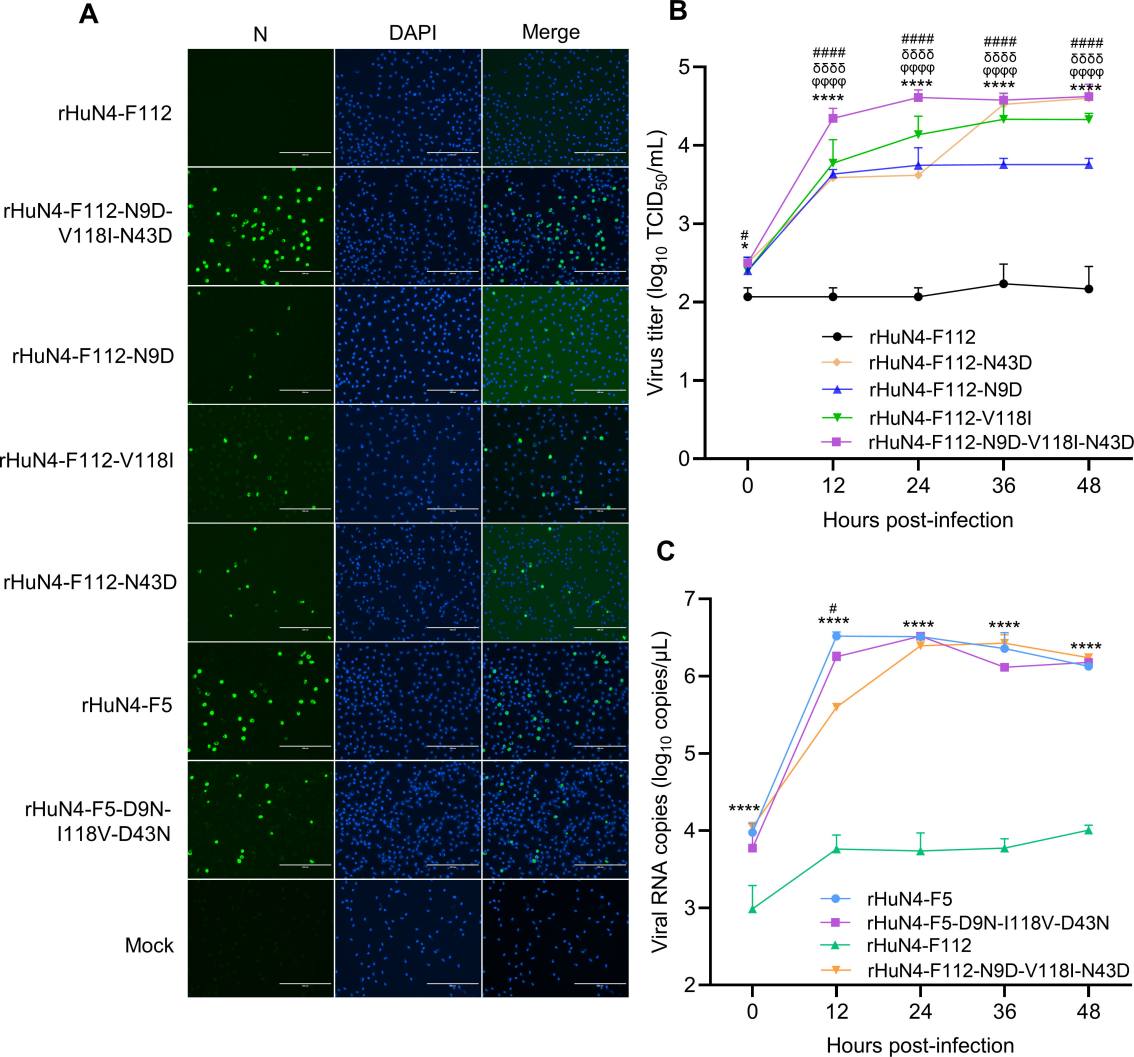
Infectivity and replication kinetics of parental and mutant strains. (**A**) The parental HuN4-F112 and mutant viruses were used to infect PAM cells at an MOI of 0.1. The cells were fixed at 24 hpi and subjected to IFA with a monoclonal antibody. (**B**) Multistep growth curves of the parental HuN4-F112 virus and mutant viruses. The viral titers were measured at 0, 12, 24, 36, and 48 hpi. Statistical analysis was performed with two-way ANOVA. Symbols indicate significant differences between groups: # for differences between HuN4-F112 and rHuN4-F112-N9D-V118I-N43D (####, *P* < 0.0001); δ for differences between HuN4-F112 and rHuN4-F112-V118I (*P* < 0.0001); φ for differences between HuN4-F112 and rHuN4-F112-N43D (φφφ, *P* < 0.0001); and * for differences between HuN4-F112 and rHuN4-F112-N9D (****, *P* < 0.0001). (**C**) Replication kinetics of the parental HuN4-F5 and rHuN4-F112 viruses and mutant viruses. # indicates differences between HuN4-F5 and rHuN4-F5-D9N-I118V-D43N (#, *P* < 0.05), and * indicates differences between HuN4-F112 and rHuN4-F112-N9D-V118I-N43D (****, *P* < 0.0001).

To determine whether the three amino acid residues are associated with PAM tropism, parental viruses HuN4-F5 and HuN4-F112, along with their mutant derivatives, were inoculated into PAM cells at an MOI of 0.1. Viral infection efficiency was assessed 24 h post-infection (hpi). IFA results revealed that HuN4-F112 failed to infect PAM cells, whereas the proliferative ability of the mutant virus carrying the N9D, V118I, and N43D mutations, designated rHuN4-F112-N9D-V118I-N43D, was restored in PAM cells (Fig. 3A). Single amino acid mutants were capable of replicating in PAM cells, but the number of infected cells was lower compared to rHuN4-F112-N9D-V118I-N43D. The multistep growth curves of HuN4-F112 and its mutant derivatives in PAM cells confirmed that the parental HuN4-F112 strain was unable to replicate efficiently in PAM cells, whereas the rHuN4-F112-N9D-V118I-N43D mutant virus replicated robustly (*P* < 0.0001). Compared with the triple mutant, the single-point mutants exhibited reduced proliferation, indicating a synergistic effect among the mutated amino acids (Fig. 3B and Fig. S1). Notably, the replication level of the rHuN4-F112-N9D-V118I-N43D mutant virus was not significantly different from that of HuN4-F5 (Fig. 3C).

### The HuN4-F112 MLV-based GP2a or GP4 revertant showed a marked increase in attachment to PAM cells

**Fig 4:**
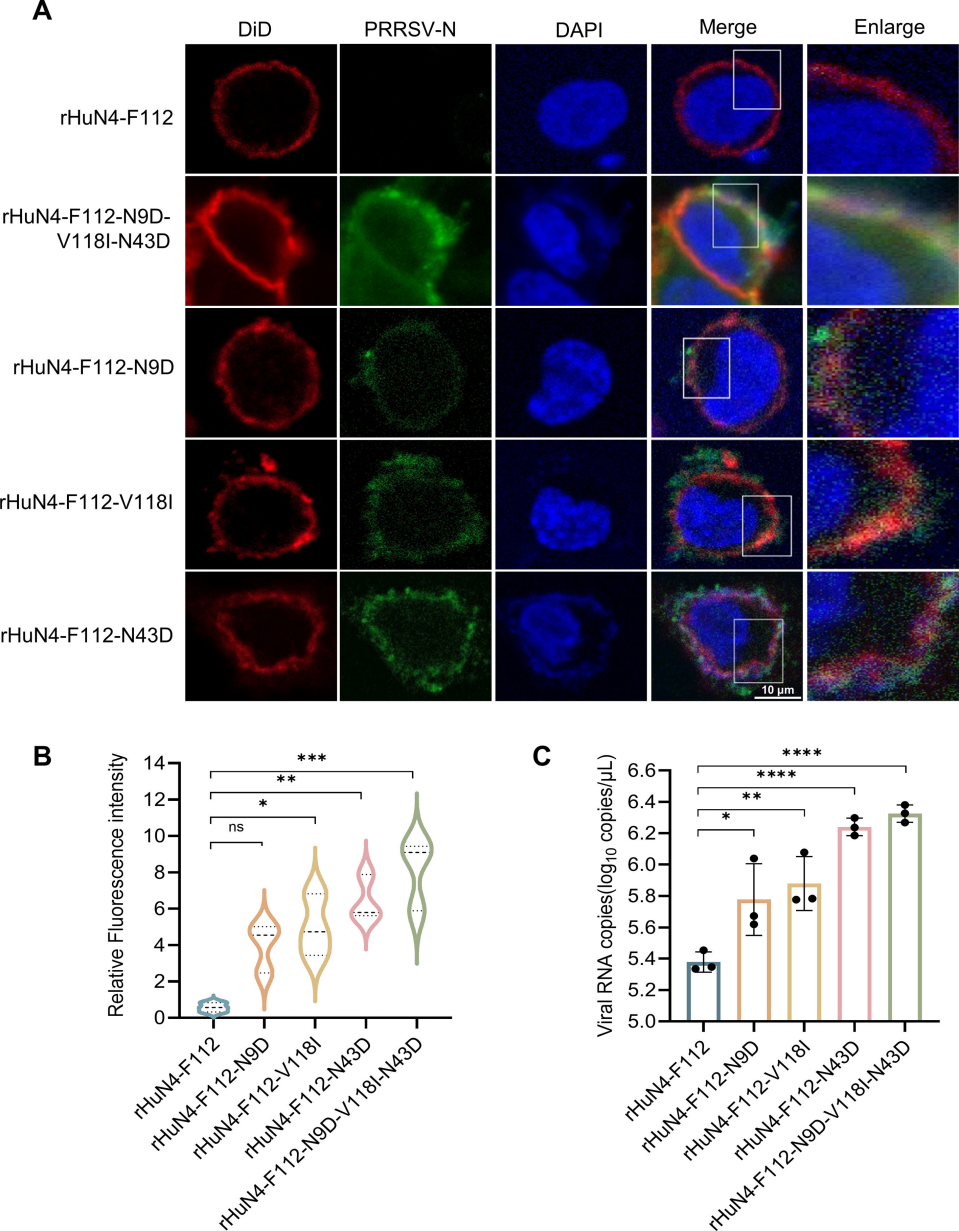
Differences in the viral attachment of HuN4-F112 and its mutants to PAM cells. (**A**) Viral attachment and cell membrane colocalization analysis. The parental HuN4-F112 and mutant viruses were inoculated into PAM cells at an MOI of 50. After 1 h of attachment at 4°C, the cells were fixed to detect the attachment status of the virus to the PAM cells. (**B**) Quantitative analysis of the fluorescence intensity of HuN4-F112 and the mutant viruses attached to the PAM cell membrane. The dotted line represents the quartiles of the violin plot data. (**C**) Quantification of PAM-attached PRRSV by RT-qPCR. ns, not significant; *, *P* < 0.05; **, *P* < 0.01; ***, *P* < 0.001; ****, *P* < 0.0001.

To determine whether the mutant amino acids in E, GP2a, and GP4 affect viral attachment, PAM cells were infected with HuN4-F112 MLV or rHuN4-F112-based mutants at an MOI of 50. The HuN4-F112 virus was not detectable on the cell membrane by IFA, whereas the rHuN4-F112-N9D-V118I-N43D mutant virus was detectable (Fig. 4A). rHuN4-F112-V118I and rHuN4-F112-N43D were also detectable. Significant differences in green fluorescence signal intensity were observed between the parental rHuN4-F112 virus and the mutants rHuN4-F112-V118I, rHuN4-F112-N43D, and rHuN4-F112-N9D-V118I-N43D (*P* < 0.05) via quantitative analysis of fluorescence intensity (Fig. 4B). The RT-qPCR results were consistent with the confocal data. Compared with rHuN4-F112, rHuN4-F112-N43D and rHuN4-F112-N9D-V118I-N43D presented highly significant increases in viral genomic copy number (*P* < 0.0001). Compared with the other three rHuN4-F112-based revertants, rHuN4-F112-N9D presented lower fluorescence intensity or viral genomic copy number (Fig. 4C).

### The HuN4-F112 MLV-based GP2a or GP4 revertant does not promote viral internalization

**Fig 5:**
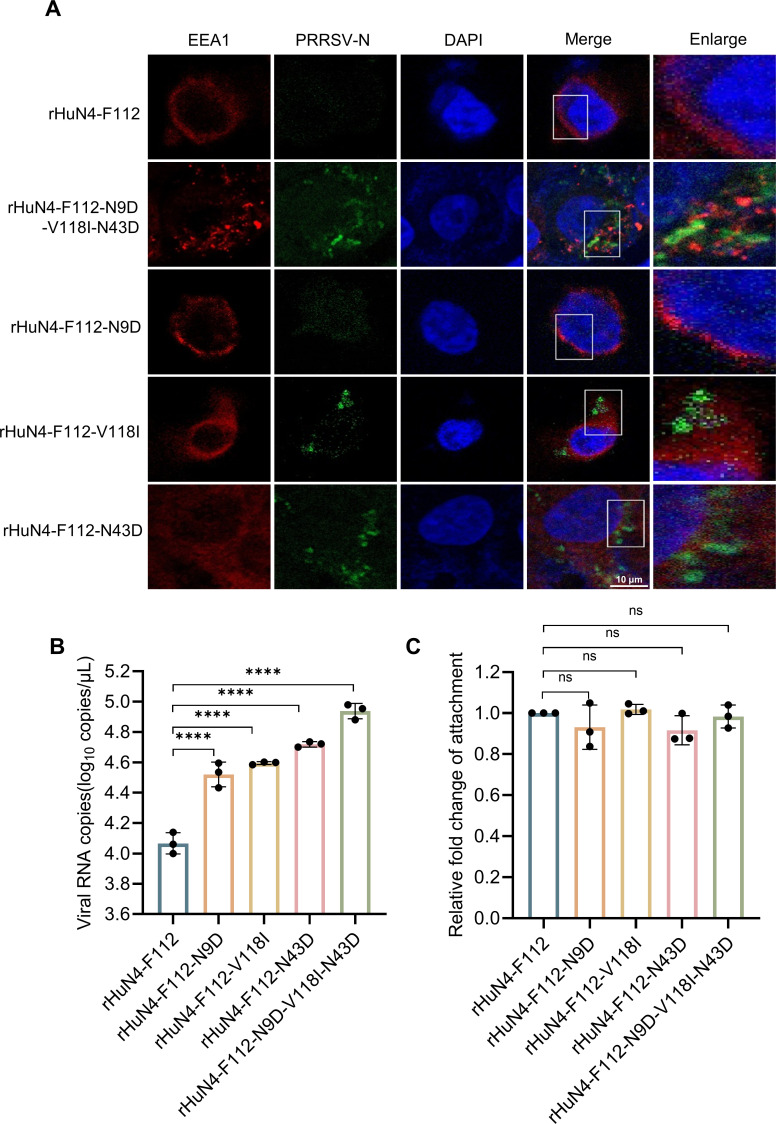
Differences in viral internalization of the HuN4-F112 virus and its mutants in PAM cells. (**A**) Confocal microscopy analysis of viral internalization. The parental HuN4-F112 and mutant viruses were inoculated into PAM cells at an MOI of 50. The internalization of the virus on PAM cells was imaged, and colocalization with EEA1 was detected. (**B**) Quantitative analysis of the internalized virus. The parental rHuN4-F112 and mutant viruses were inoculated into PAM cells at an MOI of 50. After attachment at 4°C for 1 h, the cells were washed with cold PBS, and the amount of attached virus was measured via RT-qPCR. After incubation at 37°C for 2 h, the cells were washed eight times with cold PBS at pH 1.3, and the amount of internalized virus was measured via RT-qPCR. (**C**) Ratio of internalized virus to attached virus. ns, not significant. ***, *P* < 0.001; ****, *P* < 0.0001.

To investigate whether the GP2a or GP4 revertant facilitates viral internalization, the colocalization of rHuN4-F112 mutants with the early endosome marker EEA1 was investigated (26). The mutant viruses rHuN4-F112-N9D-V118I-N43D, rHuN4-F112-V118I, and rHuN4-F112-N43D were colocalized with EEA1 (Fig. 5A). The RT-qPCR results further demonstrated that the internalization of the rHuN4-F112 mutants was significantly greater than that of rHuN4-F112 (*P* < 0.0001), especially that of rHuN4-F112-N9D-V118I-N43D, whose internalization was 10-fold greater than that of rHuN4-F112 (Fig. 5B). To eliminate the potential influence of viral attachment efficiency on internalization outcomes, we calculated the relative ratio of the internalized viral level to the attached viral level. There was no significant difference between rHuN4-F112 mutants and rHuN4-F112 (Fig. 5C), indicating that the GP2a V118I or GP4 N43D mutation does not promote viral internalization and that the change in internalization amount was due to the influence of the degree of the viral attachment process.

### GP2a- or GP4-derived small peptides can bind to PAM cells and block viral attachment

**Fig 6:**
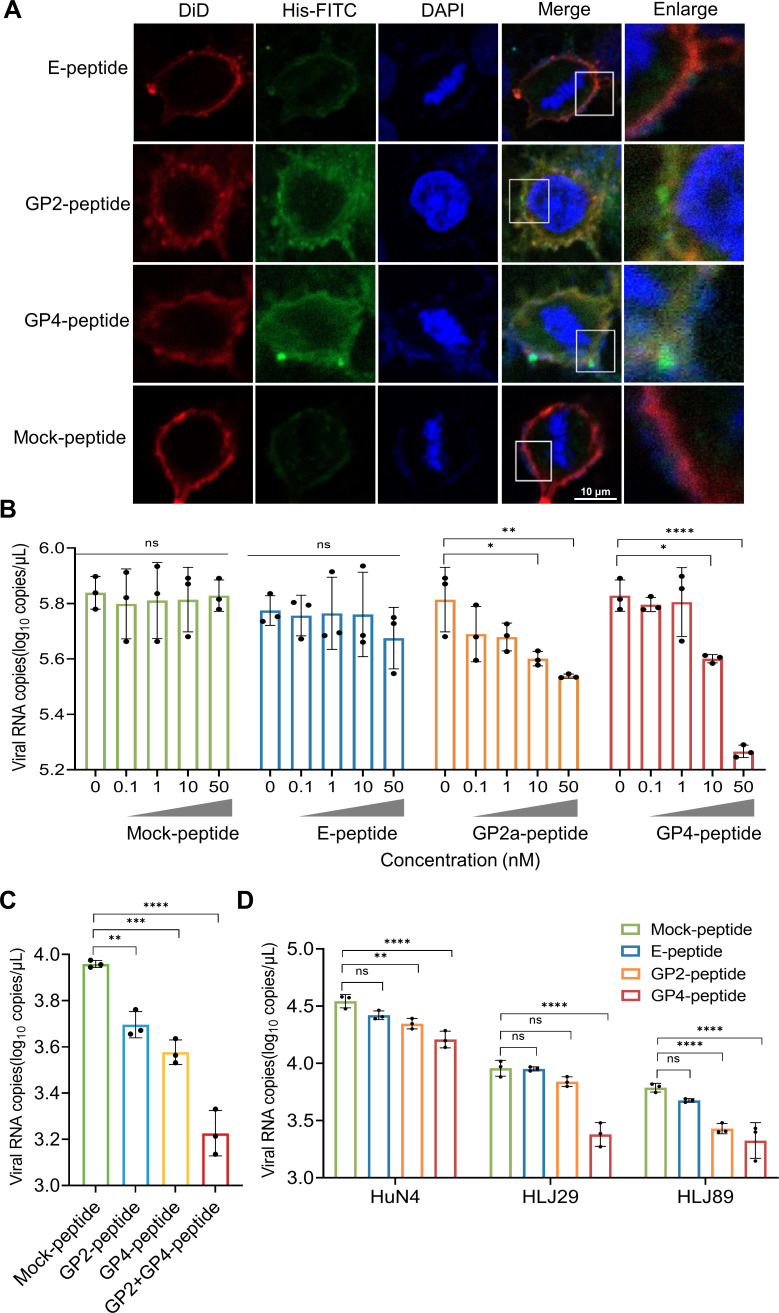
Binding of peptides to PAM cells and impact of peptides on viral attachment. (**A**) His-tagged peptides were preincubated with PAM cells at a concentration of 50 nM at 37°C for 1 h. After washing three times with PBS, the peptides bound to the PAM cells were captured. (**B**) Impact of peptides on viral attachment. Peptides were preincubated with PAM cells at concentrations of 0, 0.1, 1, 10, and 50 nM at 37°C for 1 h. rHuN4-F112-N9D-V118I-N43D was inoculated onto PAM cells at 4°C for 1 h; the cells were subsequently washed three times, and the viral load attached to the cell surface was measured via RT-qPCR. (**C**) Combinatorial impact of peptides on viral attachment. GP2a, GP4, GP2a + GP4, and mock peptides were preincubated with PAM cells at a concentration of 50 nM, and the viral load attached to the cell surface was measured by RT-qPCR. (**D**) Blocking effect on different PRRSVs. The peptides were preincubated with the PAM cells at a concentration of 50 nM at 37°C for 1 h. After washing three times, the HP-PRRSV, NADC34-like, and NADC30-like strains were inoculated. After attachment at 4°C for 1 h, the cells were washed, and the viral load attached to the cell surface was measured by RT-qPCR. ns, no significant difference. ***, *P* < 0.001; ****, *P* < 0.0001.

Three small peptides were chemically synthesized according to the sequence of rHuN4-F112-N9D-V118I-N43D. All the small peptides were 19 residues in length, with the mutated residue located in the middle, flanked by nine residues on each side. To facilitate detection, a His-tag was added to the carboxyl terminus of each short peptide. The results demonstrated that GP2a- or GP4-derived small peptides were able to bind to the surface of PAM cells and colocalize with the DiD-labeled cell membrane. In contrast, the E-peptide and the irrelevant mock peptide exhibited almost no adsorption on the PAM cell surface (Fig. 6A).

### GP2a- or GP4-derived small peptides can block viral attachment to PAM cells

To verify whether the peptides could block viral attachment, the PAM cells were preincubated with the peptides for 1 h before rHuN4-F112-N9D-V118I-N43D attachment, and then RT-qPCR was employed to quantify the number of viral copies attached to the surface of the PAM cells. The results revealed that when the peptide concentration reached 10 nM, the viral attachment was significantly lower in the GP2a or GP4 peptide groups compared to the mock peptide group (*P* < 0.05). Furthermore, the peptides effectively reduced viral attachment in a dose-dependent manner (Fig. 6B). Notably, when GP2a and GP4 peptides were combinatorially incubated with PAM cells, the viral copy number was lower than that observed in the groups treated with either peptide alone (*P* < 0.0001), indicating that the GP2a and GP4 peptides had synergistic effects (Fig. 6C). To investigate whether the peptides could block the viral attachment of different PRRSV strains, PAM cells were preincubated with the GP4 peptide at a concentration of 50 nM before attachment to wild-type PRRSV HuN4 (HP-PRRSV), HLJ29 (NADC34-like PRRSV), or HLJ89 (NADC30-like PRRSV). Compared with the mock peptide, the GP4 peptide significantly blocked the attachment of HP-PRRSV, NADC30-like PRRSV, and NADC34-like PRRSV, indicating that the peptide has broad-spectrum activity in blocking viral attachment (Fig. 6D).

### Comparison of the binding affinities of the GP2a or GP4 mutants for the CD163 receptor on PAM cells

**Fig 7:**
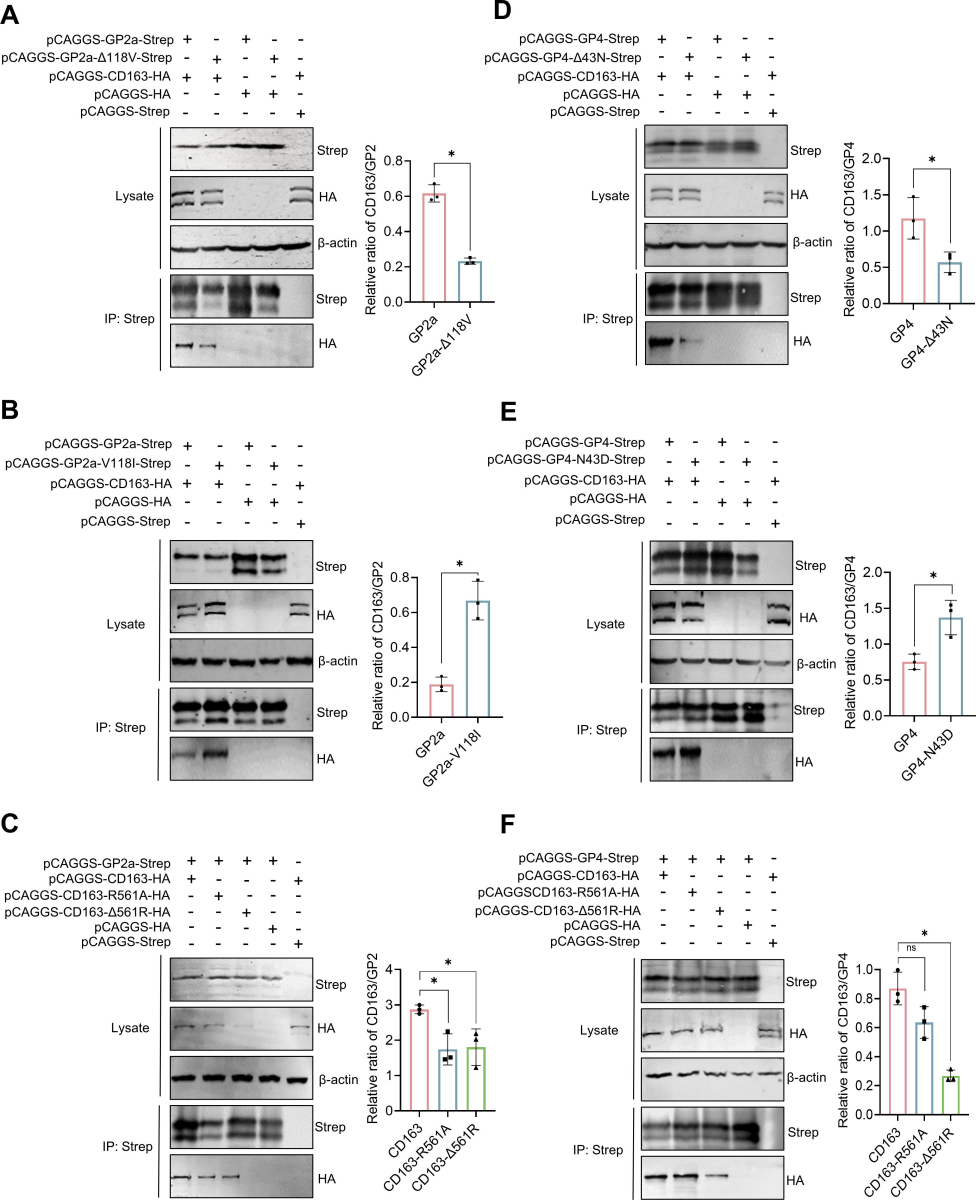
CD163 receptor binding capacity of GP2a- or GP4 mutants. (**A and B**) Co-IP assays of CD163 and GP2a containing the 118th deletion or mutation, respectively. Plasmids expressing the GP2a protein or a GP2a mutation, together with the CD163 plasmid, were cotransfected into HEK-293T cells. The intensity of the western blot bands was analyzed via ImageJ software. The strength of the interaction between CD163 and GP2a with the 118th deletion or mutation protein was evaluated. The ratio of IP-CD163 to viral protein input was used as the *y*-axis. (**C**) Co-IP assay of GP2a and CD163 containing the 561st deletion or mutation. (**D and E**) Interaction between CD163 and GP4 with the 43rd deletion or mutation, respectively. (**F**) Co-IP assay of GP4 and CD163 containing the 561st deletion or mutation. ns, no significant difference; ***, *P* < 0.001; ****, *P* < 0.0001.

To explore the mechanism by which the amino acids at positions GP2a ^118^ and GP4 ^43^ from HuN4 F112 affect virus attachment, co-IP was performed to investigate the binding affinity of GP2a or GP4 mutants for the CD163 receptor. Compared with the interaction between GP2a and CD163, the interaction between GP2a-Δ118 V and CD163 was significantly reduced (*P* < 0.05; Fig. 7A). GP2a-V118I, where the 118th amino acid changed from V in the PRRSV MLV to I in the wild-type PRRSV, significantly increased the interaction with CD163 (*P* < 0.05; Fig. 7B). The residue R561 located in the SRCR5 domain of the CD163 receptor is considered important for mediating viral infection and likely affects the binding of PRRSV to CD163 (27); thus, CD163-R561A and CD163-Δ561R are expressed. Co-IP revealed that the interactions between GP2a and CD163-R561A or CD163-Δ561 were significantly reduced (*P* < 0.01; Fig. 7C). For the GP4-Δ43 N protein, the interaction with CD163 was significantly weaker than that with GP4 (*P* < 0.05; Fig. 7D); for the GP4-N43D protein, the interaction with CD163 was significantly greater (*P* < 0.05; Fig. 7E). The interactions between GP4 and CD163-R561A or CD163-Δ561 were significantly reduced (*P* < 0.05; Fig. 7F). These results revealed that mutations or deletions at GP2a ^118^ or GP4 ^43^ affect the interaction with the CD163 receptor.

## DISCUSSION

PRRSV employs multiple receptors for cellular attachment and entry in PAM cells. Currently, the prevailing view is that the GP5/M heterodimer complex mediates viral binding to heparan sulfate and sialoadhesin receptors on PAM cells (5, 6), while the GP2a-GP3-GP4 heterotrimer mediates viral binding to the CD163 receptor (21, 28). The CD163 receptor is a crucial receptor for viral internalization, whereas heparan sulfate or sialoadhesin receptors are not (7, 15, 21). The minor envelope proteins GP2a and GP4 of PRRSV determine cellular tropism (19, 21, 29). However, the key amino acid determinants for the binding of GP2a or GP4 to the CD163 receptor in PAM cells remain undefined. In the present study, PRRSV MLVs lost their infectivity in PAM cells due to serial passaging of Marc-145 cells; however, the change in cellular tropism is still unknown.

**Fig 8:**
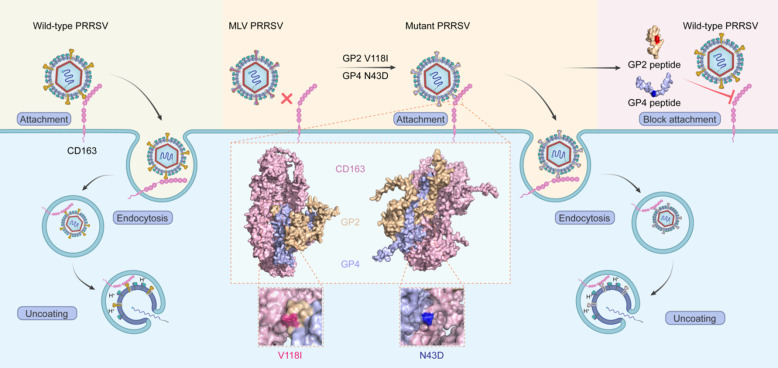
Proposed working model for PRRSV attachment and peptide blocking. GP2a and GP4 mutations influence the interaction of the virus with the CD163 receptor, thereby influencing viral attachment to PAM cells. The designed GP2a or GP4 peptides bind to the CD163 receptor in advance, thereby inhibiting viral attachment.

In this study, multistep growth curves and IFA results revealed that HuN4-F112 was ineffective at infecting PAM cells. In contrast, rHuN4-F112-V118I, rHuN4-F112-N43D, and rHuN4-F112-N9D-V118I-N43D exhibited restored infectivity to PAM cells. The attachment was enhanced in HuN4-F112 MLV-based mutants. Mechanistically, synthetic GP2a and GP4 peptides effectively blocked viral attachment across multiple PRRSV lineages by disrupting the virus-receptor interaction (Fig. 8).

The 118th amino acid in GP2a of HuN4-F112 was mutated from V to I, both of which are hydrophobic residues. In contrast, the 43rd amino acid in GP4 was mutated from N, a neutral amino acid containing an amide group, to D, a negatively charged and hydrophilic residue. This change may enhance surface exposure of the residue and facilitate receptor binding. In the HuN4-F112 strain, the 118th amino acid of GP2a and the 43rd amino acid of GP4 are exposed on the surface of the protein and located in the extracellular region, respectively (30). The R561 mutation in CD163 had differential effects on its interactions with the PRRSV structural proteins GP2a and GP4, with a more significant reduction in the interaction with GP2a. These findings suggest that R561 plays a more critical role in mediating the binding between CD163 and GP2a than it does with GP4. This finding supports the hypothesis that R561 may serve as a key contact site for GP2a during viral attachment or entry. These results provide valuable insight into the molecular determinants of virus-receptor specificity and contribute to a better understanding of the mechanisms underlying PRRSV entry into host cells.

Marc-145 cells express monkey CD163 on the cell surface. The amino acid identity of monkey CD163 is 84.8% of that of PAM cells, and both retain the SRCR5 domain required for PRRSV entry (7). Compared with that of porcine CD163, the crystal structure of monkey CD163 SRCR5 exhibited nearly identical overall folds but markedly distinct surface electrostatic potentials (31). Transfection with CD163 derived from Marc-145 cells confers PRRSV susceptibility to nonpermissive cell lines. Although CD163 is expressed in Marc-145 cells, facilitating PRRSV infection, its expression level is considerably lower than that in CD163-transfected cell lines (7). Additionally, when Marc-145 cells overexpress the porcine CD163 protein, the PRRSV-2 isolation rate increases significantly (32). These findings indicate that the efficiency of CD163-mediated entry is limited in Marc-145 cells under natural conditions. Consequently, PRRSV entry in Marc-145 cells likely relies more significantly on alternative receptor-mediated entry pathways to achieve efficient infection (25). PRRSV MLVs usually have a reduced ability to infect PAM cells after continuous passage with Marc-145 cells. Because PRRSV strongly relies on CD163 in PAM cells but has a weaker dependence on Marc-145 cells, continuous passaging of Marc-145 cells permits mutations at key amino acid residues involved in the CD163 interaction. This fundamental difference in receptor dependency drives the distinct cellular tropism of the MLV observed between PAMs and Marc-145 cells.

In a previous study, a WHE peptide (WHEYPLVWLSGY) derived from a conserved motif of CD163 SRCR5 was shown to bind to GP4 and block viral entry into PAM cells. The fragment of GP4 (C23KPCFSSSLSDIKTNTTAASGFVVLQDI50) is predicted to bind to the WHE peptide; however, this prediction has not been experimentally verified (33). In this study, the 43rd amino acid of GP4 was confirmed to have a significant influence on viral binding to the CD163 receptor. In GP2a, an I160K mutation was reported to increase viral infectivity to PAM cells (25). Additionally, 91, 97, and 98 amino acid substitutions in GP2a of PRRSV play critical roles in determining Marc-145 adaptation (23). These findings differ from the 118th position discovered in this study.

Epitope mapping revealed that GP2a contains at least three weak B-cell epitopes (positions 36–51, 117–139, and 120–142) (34) and that GP4 possesses a variable neutralizing epitope (positions 57–68) (35). The change in the 43rd residue in GP4 reduced the reaction of the peptide with PRRSV-positive sera, suggesting that the 43rd residue in GP4 is involved in the formation of an epitope (36). In this study, a peptide containing the 43rd residue in GP4 inhibited viral attachment to the CD163 receptor, suggesting that this epitope may be a potential neutralizing epitope. To date, very few neutralizing epitopes have been identified in PRRSV. In SARS-CoV-2, sera generated by immunization with recombinant protein expressing the receptor-binding motif exhibit neutralizing activities against the virus (37). GP2a at amino acid 118 and GP4 at amino acid 43 are associated with viral attachment, affecting the binding of the virus to the receptor. These sites may be targets for neutralizing antibodies that block viral infection in PAM cells.

PRRSV has continuously threatened the global swine industry, making the development of vaccines and antiviral drugs an important field. Current research indicates that blocking the binding of CD163 to PRRSV can effectively prevent viral infection. Peptides derived from the CD163 receptor directly interact with the receptor-binding regions of PRRSV proteins, thereby exerting effective neutralization, which is a major neutralization strategy (38). By interfering with the viral attachment phase, antibodies targeting the CD163 SRCR5-9 domain and the CD163 receptor have demonstrated strong affinity and antiviral activity (39). Small-molecule compounds targeting SRCR5 significantly inhibit the interaction between GP2a, GP4, and the CD163-SRCR5 domain, thereby suppressing viral infection (40). Conjugating specific nanobodies that inhibit PRRSV-targeting viral proteins with a neutralizing CD163 epitope peptide results in significant antiviral activity (41). Current research is focused on targeting CD163 motifs to prevent the virus from binding to its receptor. In this study, the GP2a peptide and GP4 peptide, which target viral sequences, bind to the receptor in advance, thereby inhibiting viral attachment and exerting broad-spectrum inhibitory effects on different lineage viruses in a dose-dependent manner by disrupting the interaction between PRRSV proteins and the CD163 receptor.

In conclusion, two important residues associated with PRRSV MLVs that disable their infectivity were identified for the first time. These two residues play critical roles in viral attachment to the CD163 receptor of PAM cells. Furthermore, viral attachment to PAM cells can be blocked by synthesized small peptides (Fig. 8). These results provide novel insights into PRRSV tropism, reveal new targets for the design of anti-PRRSV drugs or the development of neutralizing antibodies, and serve as a reference for studying the tropism of other viruses.

## MATERIALS AND METHODS

### Cells and viruses

PAM cells were prepared from the lung lavage fluid of 4-week-old specific pathogen-free (SPF) piglets and maintained at 37°C with 5% CO_2_ in RPMI 1640 medium (Sigma-Aldrich, USA) supplemented with 10% fetal bovine serum (FBS), 100 mg/mL kanamycin, and 50 U/mL penicillin. Marc-145 and HEK293T cells were grown at 37°C with 5% CO_2_ in DMEM (Sigma-Aldrich, USA) supplemented with 10% FBS. The HP-PRRSV HuN4 strain (GenBank accession no. EF635006) and its modified live vaccine HuN4-F112 strain, NADC30-like PRRSV (HLJ89 strain), and NADC34-like PRRSV (HLJ29 strain) were stored in our laboratory.

### Construction of plasmids

The infectious clone plasmids pOK-HuN4-F5 and pOK-HuN4-F112 were constructed and stored in our laboratory. Based on the infectious clones, a series of mutant viruses was constructed using primers that carried amino acid mutations or deletions (Table S1).

To probe the interaction between the GP2a/GP4 mutant and the CD163 receptor, eukaryotic expression plasmids, including pCAGGS-GP2a-Strep, pCAGGS-GP2a-Δ118 V-Strep, pCAGGS-GP4-Strep, pCAGGS-GP4-N43D-Strep, pCAGGS-GP4-Δ43 N-Strep, pCAGGS-CD163-HA, pCAGGS-CD163-Δ561R-HA, and pCAGGS-CD163-R561A-HA, were constructed via PCR and confirmed via DNA sequencing.

### Virus rescue and identification

The constructed infectious clones were transfected into Marc-145 cells to rescue the mutant viruses, as described in a previous study (42). The cytopathic effect was monitored at 4–7 days post-transfection, and the rescued viruses were identified via an immunofluorescence assay (IFA). A volume of 140 µL of the supernatant was collected, and viral RNA was extracted for RT-PCR. The PCR products were then sequenced to confirm the rescued mutant PRRSV. After three passages in Marc-145 cells, the rescued virus was utilized in subsequent viral infection assays.

### Growth kinetics of rescued viruses in PAM cells

Primary PAM cells were cultured in 24-well plates. The viruses were inoculated at an MOI of 0.01. The virus inoculum was then removed, and the cells were washed three times with PBS. Then, 2% FBS in RPMI 1640 medium was added after 1 h of incubation at 37°C. Viruses were collected at 0, 12, 24, 36, and 48 hpi. The viral copy numbers were quantified via RT-qPCR, and the TCID_50_ values of the viruses at different time points were determined in Marc-145 cells.

### Immunofluorescence assay

At 24 hpi, PAM cells were fixed with 4% polyformaldehyde, and Marc-145 cells were fixed with absolute ethanol. The fixed cells were subsequently permeabilized with 0.2% Triton X-100 for 20 min and blocked with 5% BSA in PBS for 30 min. The cells were subsequently incubated overnight at 4°C with PRRSV N monoclonal antibody (1B4) and for 1 h with anti-mouse IgG-FITC antibody. The cells were subsequently incubated with DAPI, washed with PBS, and then observed under a fluorescence microscope.

### Viral attachment assay

PAM cells in 24-well plates were infected with PRRSV, including the wild-type PRRSV HuN4 strain, the MLVs HuN4-F112, JXA1-R, TJM-F92, and GDr180, or the rHuN4-F112-related mutant PRRSV at an MOI of 50. The cells were infected at 4°C for 1 h, and then washed three times with cold PBS. Afterward, the cells were collected for viral detection via RT-qPCR or titration. For the confocal laser scanning microscopy assays, PAM cells in a chamber slide were infected with HuN4-F5-, HuN4-F112-, or HuN4-F112-based mutant viruses (MOI of 50) at 4°C for 1 h. After being washed three times with cold PBS, the cellular membrane was stained via a Far-Red Fluorescence Cell Plasma Membrane Staining Kit with DiD (Beyotime Biotechnology, China) at 37°C for 5 min. DiD (1,1′-dioctadecyl-3,3,3′,3′-tetramethylindodicarbocyanine, 4-chlorobenzenesulfonate salt) is a lipophilic carbocyanine dye featuring long hydrophobic hydrocarbon chains. Upon incorporation into the cell membrane, it undergoes lateral diffusion, allowing for the gradual staining of the entire plasma membrane.

PRRSV was labeled with an N protein monoclonal antibody, and immunofluorescence staining was performed using a protocol previously described (43, 44). Images were acquired via a confocal laser scanning microscope (model 980, Carl Zeiss).

### Viral internalization assay

PAM cells in 24-well plates were infected with HuN4-F112 or its mutant viruses at an MOI of 50 for 1 h at 4°C. The cells were washed three times with cold PBS, and then incubated at 37°C for 2 h to allow viral internalization. The cells were subsequently washed eight times with cold acidic PBS (pH 1.3) to remove any surface-bound virus. Finally, the cells were lysed to quantify the amount of intracellular viral RNA via RT-qPCR. For the confocal microscopy assays, PAM cells in a chamber slide were inoculated with HuN4-F112 or its mutant viruses, incubated at 4°C for 1 h, washed three times with cold PBS, and then incubated at 37°C for 30 min (26, 44). The fixation, permeabilization, and blocking steps for PAM cells were performed as previously described. The cells were incubated with PRRSV N monoclonal antibody (1B4) and EEA1 anti-rabbit antibody (Proteintech, China) overnight at 4°C; after being washed with PBS, anti-mouse IgG-FITC antibody (Sigma, USA) and anti-rabbit IgG-Cy3 antibody (ABclonal, China) were added for 1 h, and then the cells were stained with DAPI for 15 min. Images were acquired via confocal laser scanning microscopy.

### Coimmunoprecipitation (co-IP)

HEK293T cells were transfected with constructed point-mutant/deletion plasmids or control plasmids (3 mg/well). At 24 hpi, the cells were lysed with IP lysis buffer (Beyotime Biotechnology, China) containing PMSF (Beyotime Biotechnology, China) for 30 min on ice. The cell lysates were centrifuged at 12,000 × *g* for 10 min, and 40 µL of the supernatant was used as the input sample, mixed with 5× SDS PAGE loading buffer. The remaining supernatant was incubated with 15 µL of Magrose Strep-Tactin (Solarbio, China) at 4°C for 4–6 h. Subsequently, the beads were rinsed three times with PBS (20 min/wash) at 4°C, and the precipitates were eluted in 40 µL of lysis buffer containing 5× SDS PAGE loading buffer, followed by boiling. The samples were detected by western blotting with anti-β-actin (mouse; Proteintech, China), anti-StrepII-Tag (mouse; Proteintech, China), and anti-HA (mouse; ABclonal, China) antibodies.

### Peptide binding assay

On the basis of the sequence of rHuN4-F112-N9D-V118I-N43D, peptides encompassing the mutation sites along with flanking residues on both sides were synthesized (Table 1), and a GFP-derived peptide was also synthesized as an irrelevant mock control. Each peptide contained a C-terminal His-tag. PAM cells were seeded onto 48-well culture slides, and then incubated with peptides at 37°C for 1 h. After washing three times with PBS, the cellular membrane was stained with DiD at 37°C for 5 min. The cells were then fixed with 4% PFA for 20 min, blocked with 5% BSA in PBS for 30 min, and incubated with His-Tag antibody (mouse; Proteintech, China) overnight at 4°C. The cells were incubated with an anti-mouse IgG-FITC antibody for 1 h, and then stained with DAPI for 15 min. Images were acquired via a confocal laser scanning microscope.

**TABLE 1:** Information of the peptides and sequences

Peptide name	Peptide sequence
E-peptide	MGSMQSLFDKIGQLFVDAFHHHHHH
GP2a-peptide	EMVSRRMYRIMDKAGQAAWHHHHHH
GP4-peptide	IKTNTTAASDFVVLQDISCHHHHHH
Mock-peptide	WPTLVTTFSYGVQCFSRYPHHHHHH

### Peptide blocking assay

PAM cells in 24-well plates were incubated with different concentrations (0, 0.1, 1, 10, or 50 nM) of the peptides at 37°C for 1 h, and then washed three times with PBS. PAM cells were subsequently inoculated with different PRRSV isolates, including rHuN4-F112-N9D-V118I-N43D, HLJ29, HLJ89, and HuN4, at an MOI of 10. The cells were infected at 4°C for 1 h and then washed three times with cold PBS. Afterward, the cells were collected for viral detection via RT-qPCR.

### Statistical analysis

All statistical analyses were conducted using GraphPad Prism 8.0 software (GraphPad Software, USA). The two-way analysis of variance and *t*-tests were employed to determine statistical significance. A *P*-value < 0.05 was considered to indicate statistical significance.

## SUPPLEMENTAL MATERIAL

Fig. S1 jvi.00963-25-s0001.tiff Replication kinetics of the parental HuN4-F112 virus and mutant viruses.

Table S1 jvi.00963-25-s0002.docx Primers used in the study.

ASM does not own the copyrights to Supplemental Material that may be linked to, or accessed through, an article. The authors have granted ASM a non-exclusive, world-wide license to publish the Supplemental Material files. Please contact the corresponding author directly for reuse.
