# Supplementary figures and images for: GP2a I118 and GP4 D43 play critical roles in the attachment of PRRSV to the CD163 receptor: implications for anti-PRRSV infection targets
Source: J Virol. 2025 Aug 18;99(9):e00963-25. doi: 10.1128/jvi.00963-25 (PMC12456131; doi:10.1128/jvi.00963-25)

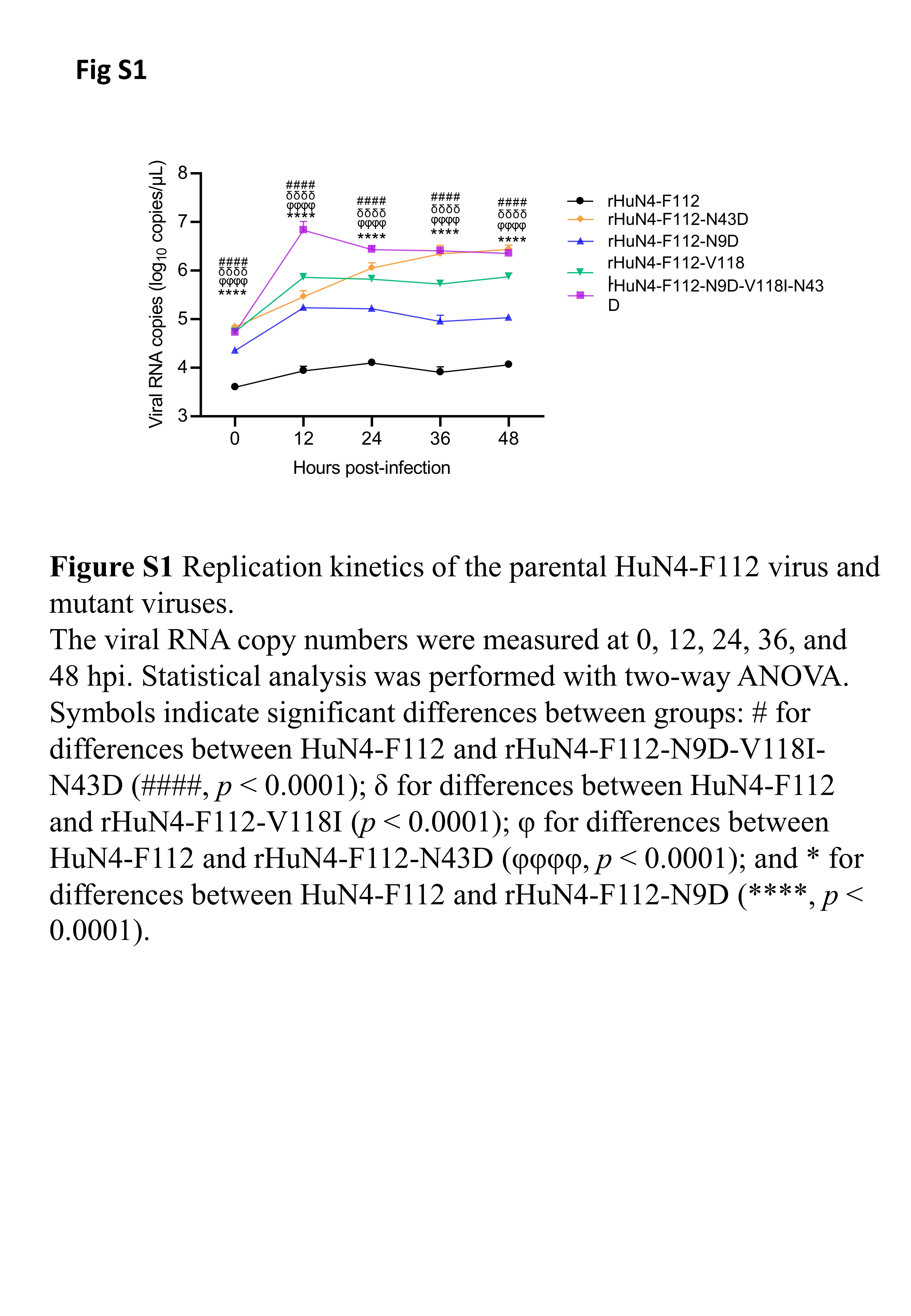

Supplement: Fig. S1 — Replication kinetics of the parental HuN4-F112 virus and mutant viruses. [file jvi.00963-25-s0001.tiff]
