# Supplementary material for: GP2a I118 and GP4 D43 play critical roles in the attachment of PRRSV to the CD163 receptor: implications for anti-PRRSV infection targets
Source: J Virol. 2025 Aug 18;99(9):e00963-25. doi: 10.1128/jvi.00963-25 (PMC12456131; doi:10.1128/jvi.00963-25)
Supplement: Table S1 — Primers used in the study. [file jvi.00963-25-s0002.docx]

Table S1 Primers used in the study

| Primer name | Sequence (5'-3') |
| --- | --- |
| AscI-F | GGATTACAATGATGCGTTTCG |
| F112-E9-M-F | AAGCCTCTTTGACAAAATTGG |
| F112-E9-M-R | CCAATTTTGTCAAAGAGGCTT |
| F112-GP2a118-M-F | TGTACCGCATCATGGATAAAG |
| F112-GP2a118-M-R | TTATCCATGATGCGGTACATT |
| F112-GP443-M-F | CGCAGCATCAGACTTCGTTGT |
| F112-GP443-M-R | ACAACGAAGTCTGATGCTGCG |
| MluI-R | GGACGCCGGACGACAAAC |
| F5-E9-M-F | GCAAAGCCTCTTTAACAAAATTGG |
| F5-E9-M-R | CCAATTTTGTTAAAGAGGCTT |
| F5-GP2a118-M-F | AATGTACCGCGTCATGGAAAAAG |
| F5-GP2a118-M-R | TTTTCCATGACGCGGTACATTCG |
| F5-GP443-M-F | CGCAGCATCAAACTTCGTTGT |
| F5-GP443-M-R | ACAACGAAGTTTGATGCTGCG |
| F112-GP2a118-D-F | AATGTACCGCATGGATAAAG |
| F112-GP2a118-D-R | CTTTATCCATGCGGTACATT |
| F112-GP443-D-F | CGCAGCATCATTCGTTGTCC |
| F112-GP443-D-R | GGACAACGAATGATGCTGCG |
| F112-GP4-Strep-F | CTCATCATTTTGGCAAAGAATTCGCCACCATGGCTGCGTCCTTTCTTTTC |
| F112-GP4-Strep-R | CTCCATCCACCGCCTCCCTCGAGAATTGCCAGTAGGATGGCAAAAAG |
| F112-GP2a-Strep-F | CTCATCATTTTGGCAAAGAATTCGCCACCATGAAATGGGGTCTATGCAAAG |
| F112-GP2a-Strep-R | CTCCATCCACCGCCTCCCTCGAGCCACGAGTTCAAAAGAAAAATTGC |
| F112-E-Strep-F | CTCATCATTTTGGCAAAGAATTCGCCACCATGGGGTCTATGCAAAGCCTC |
| F112-E-Strep-R | GCTCCATCCACCGCCTCCCTCGAGTAAGATCTTCTGTAATTGCTC |
| HA-CD163 F | ATGTTCCAGATTACGCTGAATTCGTGCTACTTGAAGACTCTGGATC |
| HA-CD163-R | TAATTAAGATCTGCTAGCTCGAGTCATTGTACTTCAGAGTGGTC |
| HA-CD163-M-F | AGTAGCACCCGCACCTGACGGGA |
| HA-CD163-M-R | TCCCGTCAGGTGCGGGTGCTACT |
| HA-CD163-D-F | CAGTAGCACCCCCTGACGGGAC |
| HA-CD163-D-R | GTCCCGTCAGGGGGTGCTACTG |
